# Ecotoxicological assessments of biochar additions to soil employing earthworm species *Eisenia fetida* and *Lumbricus terrestris*

**DOI:** 10.1007/s11356-019-04542-2

**Published:** 2019-02-22

**Authors:** Tom Elliston, Ian W. Oliver

**Affiliations:** School of Geography, Geology and the Environment, Keele University, Keele, ST5 5BG UK

**Keywords:** Earthworms, Biochar, Ecotoxicology, Soil amendment, *Lumbricus terrestris*, *Eisenia fetida*

## Abstract

Biochar is the degradation-resistant product generated by the pyrolysis of organic materials and is produced for the intended use of land application in order to promote carbon sequestration and soil improvement. However, despite the many potential benefits biochar application offers, it is important to quantify any ecological impacts that may result from soil amendment in order to avoid potentially causing negative effects upon soil biota which are crucial in the many ecosystem services provided by soil. Any impacts on earthworms in particular are important to evaluate because of their pivotal role in organic matter breakdown, nutrient cycling and soil formation. In this study, we conducted a series of ecotoxicological assays to determine lethal and sublethal (avoidance, mass change and moisture content) effects of heavy biochar applications that reflect levels that may be used in soil restoration efforts. Two earthworm species, *Eisenia fetida*, an epigeic species, and *Lumbricus terrestris*, an anecic species, were utilised as test organisms. Two types of biochar, produced from wheat straw and rice husk feedstocks, respectively, were applied to OECD artificial soil and to a natural soil (Kettering loam) at rates of up to 20% *w*/*w*. The influence of biochar application on soil porewater chloride, fluoride and phosphate concentrations was also assessed. The biochar applications induced only a subtle level of avoidance behaviour while effects on survival over a 4-week exposure period were inconsistent. However, death and physical damage to some individual earthworms at high biochar application rates were observed, the mechanisms and processes leading to which should be investigated further. Earthworm development (mean mass change over time) proved to be a more sensitive measure, revealing negative effects on *L. terrestris* at 10% and 20% (*w*/*w*) wheat biochar applications in OECD soil and at 20% (*w*/*w*) applications of both biochars in Kettering loam. The moisture content of *E. fetida* remained remarkably consistent across all treatments (~ 82%), indicating that this is not a sensitive measure of effects. The high rates of biochar application resulted in increased chloride (2 to 3-fold) and phosphate (100-fold) concentrations in simulated soil porewaters, which has important implications for soil fertility and production but also for environmental management.

**Electronic supplementary material:**

The online version of this article (10.1007/s11356-019-04542-2) contains supplementary material, which is available to authorized users.

## Introduction

Biochar is the carbonaceous residue created during the pyrolysis, i.e. thermal decomposition, of biomass under low oxygen conditions (Lehmann and Joseph 2009) and is being increasingly produced and incorporated into soil because of its potential to sequester carbon and thus mitigate climate change through increasing the long-term storage of carbon in soils (e.g. McHenry 2009). Biochar applications to land are also being promoted by many voices because it can additionally offer a host of other benefits to soils; such benefits may include increasing the recalcitrant organic matter content of soil which consequently decreases bulk density and increases porosity, water holding capacity, hydraulic conductivity and cation exchange capacity (Huang et al. 2013). Biochar has also been identified as having potential use in remediation and restoration of degraded and contaminated soils not only because of the benefits outlined above but also because it can potentially bind, or otherwise render inactive, contaminants and toxic constituents (Beesley et al. 2011; Houben et al. 2013; Rinklebe et al. 2016). Nevertheless, the wider soil ecosystem impacts of biochar addition need to be considered and evaluated, particularly as biochar addition is effectively permanent, lasting for thousands of years, on account of its recalcitrance (Lehmann and Joseph 2009; Lone et al. 2015). The effects of biochar applications on earthworms have received very limited attention (e.g. Weyers and Spokas 2011; Malev et al. 2016) and are an important research gap considering the great importance of this organism group to soil health and ecosystem processes. Indeed, earthworms have long been recognised as ecosystem engineers, playing an essential role in organic matter decomposition, nutrient cycling, pore creation and soil formation (e.g. Darwin 1881). Moreover, earthworms are a food source for a range of mammals, birds, reptiles and amphibians, and thus, for all of these reasons, it is important to avoid causing alterations to their behaviour or to their populations in order to maintain soil and ecosystem functionality. The aim of this study, therefore, was to investigate the ecotoxicological effects of biochar addition on two earthworm species of differing ecologies, *Eisenia fetida*, an epigeic species, and *Lumbricus terrestris*, an anecic (deep burrowing) species. Lethal and sublethal endpoints were examined in order to identify a range of effects resulting from soil amendment with biochar.

## Methods

### Soils and biochars

The terrestrial ecotoxicology assays in this study were conducted in a natural soil, Kettering loam (Kettering, Northamptonshire, UK, supplied by Boughton Ltd., www.boughton.co.uk) and in an OECD artificial soil constructed in the laboratory. Kettering loam was selected because it is known to be suitable for a range of earthworm species and has been employed previously in multiple earthworm studies (Brinza et al. 2014; Davies et al. 2003; Langdon et al. 2003; Lowe and Butt 2005; Lowe et al. 2016). Moreover, the characteristics of Kettering loam are well established (typically pH 6.8–7.2; clay-silt-sand = 24%-18%-58%; organic content 5–7%) (Brami et al. 2017; Lowe et al. 2016). Kettering loam was dried and sieved to 2 mm prior to use. The OECD artificial soil was assembled in accordance with OECD guideline 222 (OECD 2016) and comprised ~ 70% sand obtained from Borne Amenity Ltd., Kent, UK, sieved to 2 mm, 20% kaolin clay, 10% dried sphagnum peat and a small percentage of crushed calcium carbonate (agricultural lime grade) to increase the soil pH from its initial measured pH of 5.8 to a value within the range specified by the OECD protocol and closer to that of the Kettering loam. The OECD soil was selected to facilitate comparison with the many other ecotoxicology tests conducted with this soil (Feng et al. 2015).

Rice husk and wheat straw biochars were purchased from the UK Biochar Research Centre, Edinburgh, UK. Both biochars had been produced by pyrolysis at 550 °C and were selected because they are ‘mid-range’ in terms of the pyrolysis temperature at which biochars are generally made and thus can be considered in some respects to be representative of a range of commonly produced biochars. Also, their feedstocks are widely available agricultural waste products and so are the kinds of materials likely to be used in soil remediation and recycling/ environmental management schemes employing biochar applications. Before use in experiments, the biochars were ground into a fine powder (< 1 mm) using a mortar and pestle. The pH of the biochars (determined in 1:5 solid/deionised water slurries) was 10 and 10.5 for wheat straw and rice husk, respectively, and thus were alkaline as is typically reported for similar biochars (Beesley and Marmiroli 2011). A visual inspection of biochar surfaces was conducted through examination under a Hitachi TM-3000 scanning electron microscope (SEM) after further finely grinding subsamples and compressing into pellets and mounting on a stub using Leit-C conducting carbon cement. The SEM scans were conducted at ×500, ×1000, ×2500 and ×5000 magnification.

The organic matter content (OM%), pH and water holding capacity (WHC) of the soils and the soil + biochar treatments (described in later sections) were determined, with OM% measured by loss on ignition at 450 °C and pH measured in 1:5 solid/deionised water suspensions using a JENWAY 3510 glass pH electrode probe and meter. Soil WHC was measured by fully saturating 100 g with 100 mL deionised water, then allowing it to drain until all dripping stopped and the amount of liquid remaining in the soil calculated.

### Earthworms

*Lumbricus terrestris* and *Eisenia fetida* were purchased from Yorkshire Worms (www.yorkshire-worms.co.uk). Field collection of earthworms was decided against because of (i) the large number required (> 1000 individuals) and the time and labour that would be involved in their collection, (ii) the greater genetic and physical attribute consistency within a population of hand-reared organisms compared with field collected specimens, (iii) the risk of misidentification when dealing with large numbers and (iv) the potential for differing previous land management practices (including pesticide use) across any fields sampled that may influence earthworm behaviour and survival during the ecotox assays. Between acquisition and deployment in the assays, *E. fetida* were maintained in a moistened substrate of bonsai compost, coir fibre and peat, whereas *L. terrestris* were kept in a clean commercial topsoil mixed with peat. Earthworm cultures received additional feeding of ground oats and, occasionally, lettuce leaves.

### Earthworm avoidance assay

Avoidance assays were conducted with *E. fetida* according to the two-chamber method stipulated under ISO protocol 17512-1 (ISO 2008). The test vessels used had dimensions 15 × 15 × 10 cm (height × length × depth) and were equipped with removable vertical central partitions that divided them into two equal chambers (Howells et al. 2018). Into each vessel, 250 g non-amended (i.e. untreated) soil was placed in one chamber and an equal mass of treated soil was placed in the other. Control vessels, with untreated soil in both chambers, were also established. Before placement in the vessels, the soils had been moistened to 60% WHC. The treatments imposed on each soil were 5%, 10%, 15% and 20% biochar (*w*/*w*; *n* = 3), intended to reflect heavy biochar addition rates tested elsewhere (Li et al. 2011; Major et al. 2010), and that may be implemented in soil remediation and restoration efforts (Kosolsaksakul et al. 2018).

Upon commencement of the assay, the central partitions were removed and ten adult earthworms with visible clitellum introduced. A perforated transparent cover was fitted to prevent escape, and the vessels were left for 48 h after which the partitions were re-inserted and the soils removed and the number of earthworms present in each chamber counted. Avoidance behaviour was expressed in terms of the percentage of the earthworms found in the control soil chamber (Eq. 1).

1 $$ \mathrm{Avoidance}=\mathrm{Percentage}\ \mathrm{in}\ \mathrm{control}\ \mathrm{chamber}-50 $$

### Earthworm survival and development (mean mass change)

The effects of biochar application on earthworm survival and development (mass gain or loss) were examined following OECD protocol 222 (OECD 2016), using both *E. fetida* and *L. terrestris* (separately) in each of the test soils. Biochar application rates of 0%, 10% and 20% (*w*/*w*) were imposed (*n* = 3), with all soils and soil + biochar treatments moistened to 60–70% WHC. Plastic pots (660-mL capacity) were used as test vessels, and these were wrapped in aluminium foil to prevent lateral intrusion of light (Hund-Rinke and Wiechering 2001). The mass of soil or soil + biochar placed in the pots was equivalent to 250 g dry material. Five adult earthworms (visible clitellum) that had been rinsed with deionised water, patted dry with paper towelling and weighed were introduced to each pot. Approximately 2.5 g ground oatmeal was added to the surface of each pot to serve as food source, with a further serving provided once a week thereafter. After 14 days, the soil and earthworms were temporarily removed from their pots to facilitate earthworm survival counts and mass change determination after rinsing and drying of the earthworms. The soil and earthworms were then returned to the pots for a further 14 days, after which the final survival count and mass change measurements were recorded. Verification of survival was achieved through gentle physical stimuli with a blunt object (a pencil), and any signs of physical damage in the earthworms were noted.

At completion of the assay, the *E. fetida* specimens were further examined to investigate any impacts of biochar addition on earthworm moisture content. To achieve this, the *E. fetida* were maintained on moist filter paper for 24 h to allow depuration (Arnold and Hodson 2007) and were then rinsed in deionised water, euthanised by freezing, dried in an oven and the dry mass recorded.

An attempt was also made to examine how biochar addition affected water-soluble anions in treated soils, specifically chloride, fluoride and phosphate concentrations, to determine whether any such effects correlated with earthworm behavioural or physical effects observed. To do this, Kettering loam soils from the survival assay with *L. terrestris* were used to generate simulated porewater (Ardestani and van Gestel 2013; Ma et al. 2006) by saturating 25 g of recovered soil with 20 mL deionised water for 72 h before centrifugation for 20 min and filtration of the supernatant through 0.45-μm syringe filters. The filtered supernatants were then analysed by ion chromatography (Dionex ICS 1000) using certified reference solutions for construction of calibration curves.

### Statistical analysis

The data were examined for statistical differences amongst treatments using analysis of variance (ANOVA), *t* tests and, in cases where data were not normally distributed, Mann-Whitney and Kruskal-Wallis tests. All statistical tests were conducted using Minitab software.

## Results and discussion

### Soil and biochar properties

The pH of the Kettering loam and artificial OECD soil was well matched, both having values measured at ~ 7.6 (Table 1). Addition of rice biochar had negligible influence on soil pH in either soil whereas addition of wheat biochar substantially increased soil pH, particularly at the 20% *w*/*w* addition rate and especially in the OECD soil where the pH rose above 9.1 (Table 1). Biochar addition also substantially increased the WHC of the soils, increasing it by ~ 50% at the highest rate applied (Table 1). The organic matter content measurements confirmed anticipated values of 6.13% ± 0.08% for Kettering loam and 15.1% ± 0.74% for the OECD soil. Scans under SEM suggested that wheat biochar had a more defined pore structure than that of the rice biochar (Supplementary Information Figs. S1 and S2).

**Table 1:** Soil pH and water holding capacity (mean ± standard error)

Soil	Parameter	Untreated soil	Wheat biochar		Rice biochar	
			10%	20%	10%	20%
OECD	pH^a^	7.64 ± 0.22	8.61 ± 0.20	9.19 ± 0.30	7.67 ± 0.22	7.55 ± 0.43
Kettering		7.62 ± 0.16	8.09 ± 0.21	8.84 ± 0.03	7.79 ± 0.50	7.53 ± 0.07
OECD	WHC (mL/100 g)	58	nd	76	nd	81.8
Kettering		64	nd	97.6	nd	90.2

*WHC* water holding capacity, *nd* not determined

^a^Values measured at the end of the survival assay (i.e. after 4 weeks of equilibration)

### Earthworm avoidance

**Fig. 1:**
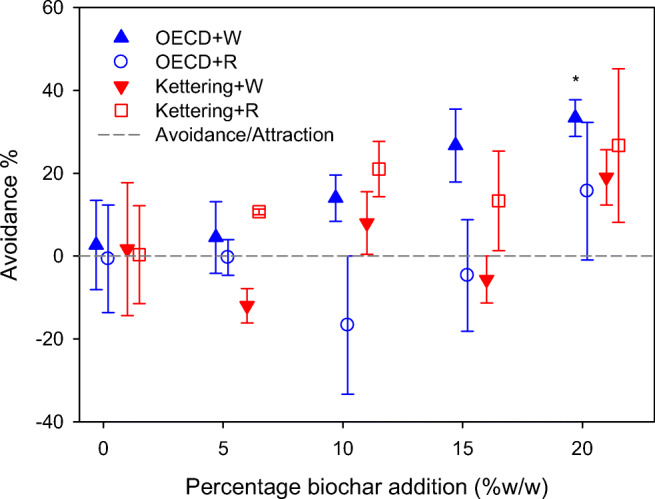
Earthworm (*Eisenia fetida*) avoidance of OECD artificial soil and Kettering loam amended with wheat (W) or rice (R) biochar. Error bars indicate standard error about means. The asterisk symbol indicates a significant difference from control in the OECD soil + wheat biochar treatment at the maximum application rate

In compliance with the validity criteria for the test protocol, no avoidance behaviour was exhibited in the controls of the avoidance assay with *E. fetida* (Fig. 1). A linear trend of avoidance was apparent for OECD soil amended with wheat biochar (i.e. linear equation: Avoidance % = 1.67 × biochar % − 0.467; *R*^2^ 0.958), with the 33% avoidance maximum exhibited at the highest treatment level imposed being statistically significant in terms of difference from the control (*t* test *t* > 0, *p* = 0.039, Fig. 1). The other treatment/ soil combinations showed little pattern, exhibiting considerable intra-replicate variability. A much more pronounced avoidance behaviour by *E. fetida* was reported by Li et al. (2011) when biochar produced from apple wood sawdust was added at 10% and 20% (*w*/*w*) to a soil closely matching the OECD soil used in the present study, indicating that biochar of differing feedstocks and production conditions can have differing impacts on earthworm behaviour. The avoidance behaviour observed here was also less pronounced than that described for *E. fetida* introduced to a soil amended with 20% (*w*/*w*) aluminium water treatment residuals, for which an avoidance rate of ~ 50% was reported (Howells et al. 2018).

### Earthworm survival

**Fig. 2:**
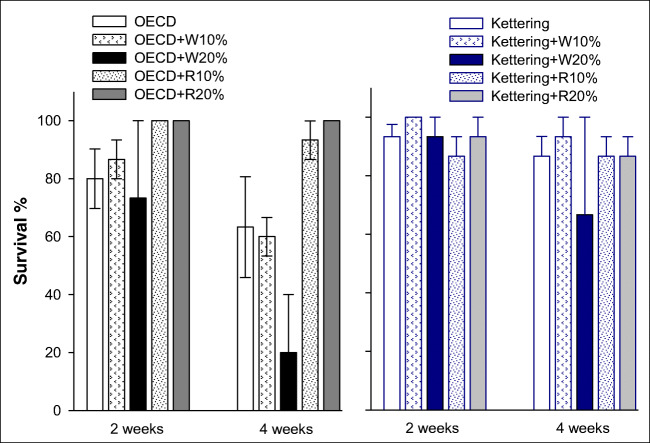
Earthworm (*Lumbricus terrestris*) survival in OECD artificial soil and Kettering loam amended with wheat (W) or rice (R) biochar at 2 and 4-week exposures. Error bars indicate standard error about means

**Fig. 3:**
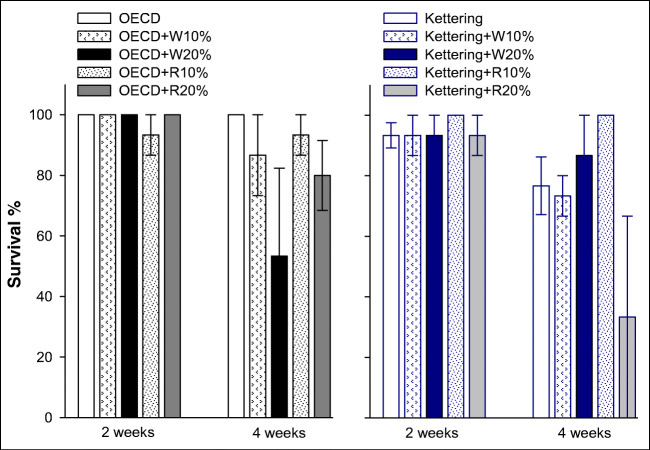
Earthworm (*Eisenia fetida*) survival in OECD artificial soil and Kettering loam amended with wheat (W) or rice (R) biochar at 2 and 4-week exposures. Error bars indicate standard error about means

After 2 weeks of exposure, there were no impacts on survival at any of the biochar treatment levels for either of the earthworm species tested (Figs. 2 and 3). By 4 weeks, there appeared to be an effect in the 20% wheat biochar treatment in the OECD soil for *L. terrestris*; however, due to the sample size and high intra-replicate variability in that treatment (replicates had values of 0%, 60% and 0% survival), the reduction was not quite identified as being significantly different from the control (*p* = 0.087). The 20% wheat treatment in the Kettering loam soil also produced replicates with considerable variability (Fig. 2), but overall, the result for that treatment was not significantly different from the control. A comparable pattern was observed for *E. fetida*, with an apparent reduction after 4 weeks of exposure in the OECD soil + 20% wheat treatment (Fig. 3). However, high intra-replicate variability for this treatment (replicates of 0%, 60% and 100% survival) meant that statistically significant differences from the control were not identified. In Kettering loam soil, the 20% rice biochar application had a lower mean survival percentage for *E. fetida* (Fig. 3), but again, high variability within this treatment (replicates with 0%, 100% and 0%) made identification of statistically significant differences impossible. A greater number of replicates would have addressed this issue; hence, a recommendation can be made for future research involving earthworm survival studies that at least four replicates per treatment be maintained in order to raise the statistical power of the test. Nevertheless, the data generated do suggest that the 20% biochar application rates applied are on the cusp of reaching a threshold value where significant effects might occur; such an inference would be in keeping with the results reported by Anyanwu et al. (2018), who tested the effects of rice husk biochar applications on *Eudrilus eugeniae* (an epigeic earthworm species comparable to *E. fetida*) and found that statistically significant effects on survival occurred at applications of 25% and 50% *w*/*w* but not at 10%. Liesch et al. (2010) also found no significant effects on survival of *E. fetida* at application rates of pine chip biochar equivalent to 10% *w*/*w*. However, the Liesch et al. study did report effects in treatments with biochar generated from poultry litter even at modest rates (~ 5% *w*/*w*), indicating that biochars from different feedstocks can have differing thresholds for effects on earthworm survival.

**Fig. 4:**
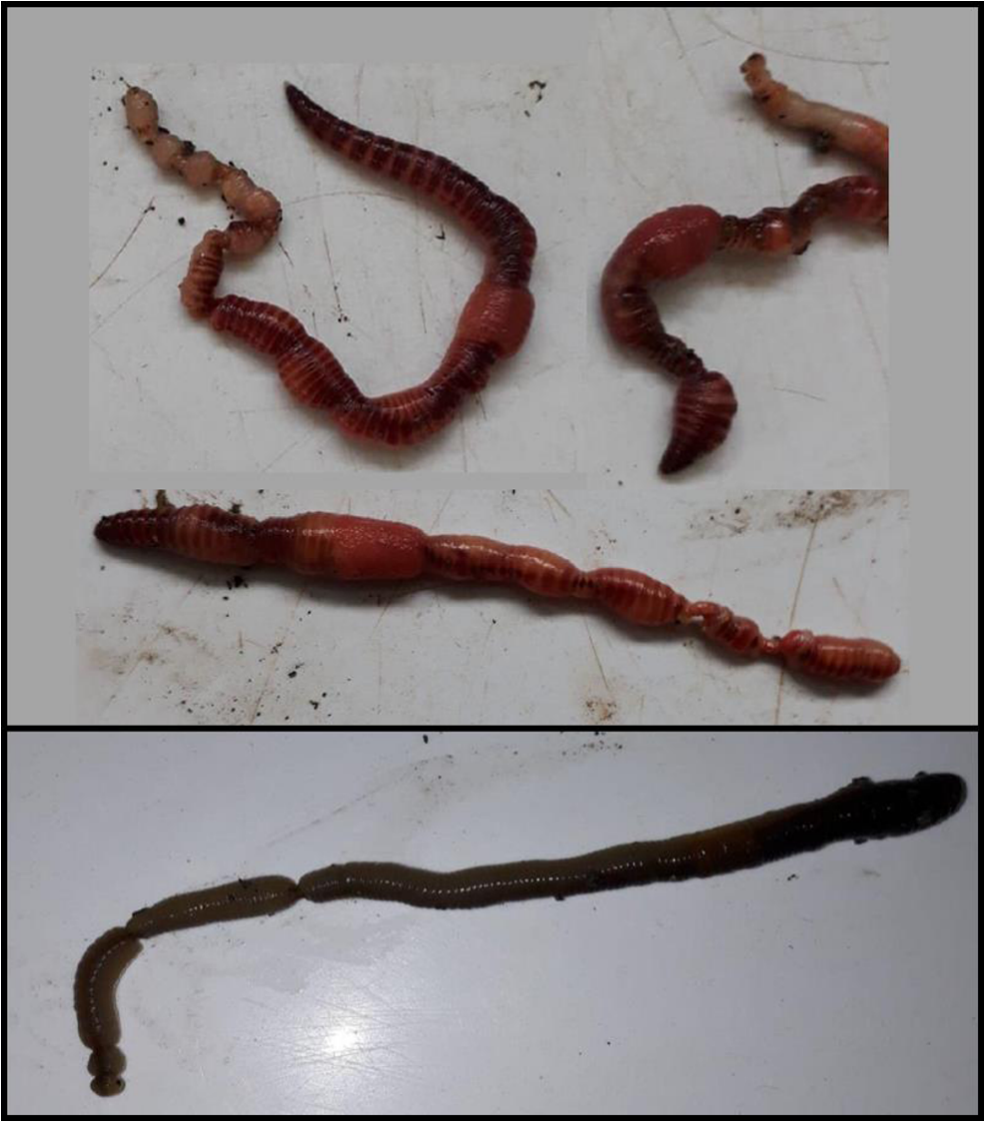
Physical damage to earthworms (*Eisenia fetida*, upper panel, and *Lumbricus terrestris*, lower panel) observed in some individuals in the high biochar application rate treatments

The wide variability in survival between the replicates of some of the high biochar application rate treatments was also reflected in the physical condition observations made, as in multiple replicates across the high biochar addition treatments there were individual earthworms that appeared badly damaged (Fig. 4). The level of damage was inconsistent within and between replicates, making it difficult to draw definitive conclusions. Nevertheless, there were clearly some serious adverse effects evident that have not been widely reported in the literature. One previous study (Malev et al. 2016), however, did similarly find physical damage to *Eisenia andrei* earthworms exposed to high, but agronomically feasible, soil applications of biochars generated from wine tree cuttings and hardwood feedstocks, with the earthworms having developed a lumpy and irregular shape and darker coloration. The physical damage may have been caused by internal or external abrasion caused by biochar particles, and the elevated soil pH resulting from high biochar applications may have also been a factor. Other possibilities should also be considered, even if the parameters were not measured in this study and so no evidence can be presented; for example, it has been found that biochar addition can raise polycyclic aromatic hydrocarbon (PAH) concentrations in treated soils and in earthworms residing in them (Malev et al. 2016), which can have a direct toxic effect. Further research is needed to determine the processes and mechanisms responsible for the physical damage.

### Earthworm development (mass change)

**Fig. 5:**
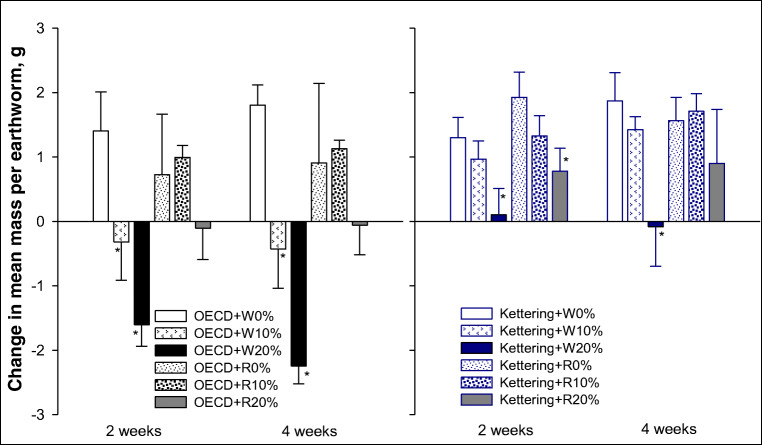
Earthworm (*Lumbricus terrestris*) development (mean change in average mass) in OECD artificial soil and Kettering loam amended with wheat (W) or rice (R) biochar at 2 and 4-week exposures. Error bars indicate standard error about means. The asterisk symbol indicates significant difference from control

For *L. terrestris*, wheat biochar additions to OECD soil resulted in significant mass loss (i.e. loss of condition) at both 10% and 20% application rates, and this was consistent at both the 2 and 4-week stage (Fig. 5). Rice biochar addition to the OECD soil did not produce results significantly different from the untreated controls; however, there was considerable variability in the controls of the rice biochar addition treatments that made any subtle effects difficult to detect (Fig. 5). In Kettering loam, the 20% application rates for both wheat and rice biochar treatments reduced mean mass after 2 weeks (Fig. 5), whereas after 4 weeks, the differences were only statistically significant for the 20% wheat biochar treatment. The development (mass change) results for *L. terrestris* indicate that this is a much more sensitive assay than the survival test, i.e. it clearly identified statistically significant effect thresholds within the biochar application rates tested whereas the survival assays were only able to provide indicative values for tipping points. This is an important conclusion and should be taken into account when ecotoxicology studies are planned and conducted with an aim to assess the effects of soil amendments such as biochar on earthworms.

**Fig. 6:**
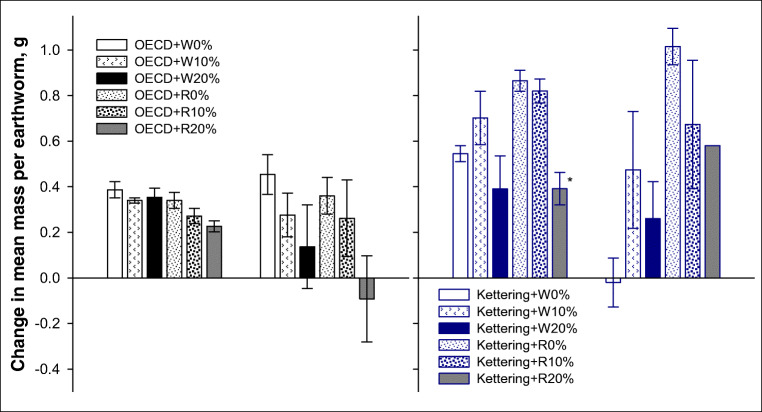
Earthworm (*Eisenia fetida*) development (mean change in average mass) in OECD artificial soil and Kettering loam amended with wheat (W) or rice (R) biochar at 2 and 4-week exposures. Error bars indicate standard error about means. The asterisk symbol indicates significant difference from control

For *E. fetida*, none of the treatments in OECD soil had detectable statistically significant effects on mean mass, but there was an apparent trend towards a reduction at the 4-week stage in the 20% biochar treatments (Fig. 6). In Kettering loam, after 2 weeks, significant reductions in mean mass were observed in the 20% rice biochar treatment (Fig. 6). The effect appeared to remain consistent for that treatment after 4 weeks, but this could not be verified statistically because only one replicate pot from this treatment had surviving earthworms to determine mean mass for (see survival results above). There was an unexpected reduction after 4 weeks in the mean mass of the control *E. fetida* earthworms for the wheat biochar treatments in Kettering loam (Fig. 6) which, together with the wide variability in the other wheat biochar treatment results in this soil at the 4 week point, makes further interpretation difficult. The reductions in mean mass observed for earthworms in some of the biochar treatments support earlier findings, e.g. Li et al. (2011) found that 10% and 20% biochar additions caused significantly more weight loss than that observed in control soils (earthworms were not fed during that study), while Gomez-Eyles et al. (2011) found that 10% *w*/*w* additions of deciduous hardwood-derived biochar also caused significantly greater weight loss in *E. fetida* compared with untreated soil at both exposure time periods assessed (28 days and 56 days).

Earthworm (*E. fetida*) moisture content was remarkably consistent across all treatments at the end of the 4-week experiment (surviving individuals assessed only). Despite differences between treatments and replicates in terms of number of survivors, mass gain/loss and visually inspected condition, all survivors had a moisture content of ~ 82% (Table 2). This is in complete agreement with the findings of Hartenstein et al. (1980) who determined that, independently of size or live mass, moisture content in the *E. fetida* they examined was 81.8% ± 7.7%. This indicates that, while moisture content is essential, it is not a sensitive measure of stress or health in *E. fetida* because the species appears to have a very strong capacity for moisture content homeostasis. It would be interesting to determine whether this was also the case for *L. terrestris*.

**Table 2:** Earthworm (*Eisenia fetida*) moisture content after the survival assay (mean ± standard error)

Treatment	0%	10%	20%
Kettering + W	0.85 ± 0.01	0.84 ± 0.00	0.82 ± 0.01
Kettering + R	0.83 ± 0.00	0.85 ± 0.02	0.84
OECD + W	0.83 ± 0.01	0.79 ± 0.08	0.80 ± 0.02
OECD + R	0.82 ± 0.00	0.83 ± 0.01	0.80 ± 0.01

### Soluble anions in simulated soil porewater

Addition of rice biochar to the Kettering loam soil had a strong, significant effect on the soluble chloride determined in simulated porewaters generated following the survival assay. At both application rates imposed, rice biochar increased the chloride concentration 2 to 3-fold (Table 3). Wheat biochar also increased the chloride concentration, but there was much more variability amongst the replicates of the wheat biochar treatments. None of the biochar treatments affected the fluoride concentrations; however, the phosphate concentration was greatly increased (~ 100-fold) in the 20% application rate of each biochar type. The observation that both wheat and rice biochars increased the water-soluble chloride and phosphate in treated soils but that significant reductions in mass of earthworms were only noted in wheat biochar treatments (and only at 20% application rate) indicates that the increase in soluble chloride and phosphate did not in itself directly impact upon the earthworms (i.e. otherwise, effects would have been observed in both cases, if the increased soluble anions were a causative factor). Nevertheless, the effects on chloride concentrations are important because it is well known that chloride ions, when in sufficient abundance, can increase the mobility of toxic elements such as Cd that may be present in the soil (Smolders and McLaughlin 1996). This will be a particularly important consideration to take into account for scenarios in which biochar is used to remediate contaminated soils (Beesley et al. 2011; Kosolsaksakul et al. 2018). The effect on salinity (and electrical conductivity) associated with increased chloride concentrations is also important to consider because this can impact the suitability of the soil environment for microbes, plants and soil fauna. Any change to the concentration of phosphate in soil porewater is very important in terms of plant fertility and primary productivity; hence, the significant increases observed here in the 20% biochar application rates warrant further exploration. This includes examination of negative aspects, because as well as being a potential positive, any increase in soluble phosphate in biochar-treated soils could also be a potential cause for concern in terms of possible increased eutrophication risk to nearby water bodies. It is well established that addition of biochar can affect P and N cycles in treated soils by stimulating microbial activity and providing reactive surfaces upon which exchange processes can occur; however, the extent and direction of effects are highly variable and are dependent upon soil-biochar interactions dictated by the specific soils and biochars involved (Gul and Whalen 2016). Mukherjee and Zimmerman (2013) found that P release from biochars was linked to biochar volatile matter and ash content as well as functional group density. Therefore, the increased phosphate concentrations observed here in porewaters of treated soil may be linked to constituents of the biochars themselves and/or to accelerated microbial activity and greater microbial diversity following biochar addition that can influence nutrient availability, as has been noted and discussed elsewhere (Lehmann et al. 2011; Xu et al. 2016).

**Table 3:** Soluble anion concentration (chloride, fluoride and phosphate, mg/L, mean ± standard error) in simulated porewaters from Kettering loam soil treated with wheat or rice biochar

Parameter	Untreated	Wheat biochar		Rice biochar addition	
	(0%)	10%	20%	10%	20%
Chloride	53.7 ± 7.4	126.6 ± 29.1	156.8 ± 51.1	121.9 ± 5.1*	185.1 ± 10.5*
Fluoride	0.82 ± 0.05	0.79 ± 0.02	0.94 ± 0.04	0.68 ± 0.06	0.54 ± 0.02
Phosphate	0.11 ± 0.07	0.62 ± 0.21	12.43 ± 3.79*	1.36 ± 0.71	8.93 ± 1.25*

*Significantly different from control

## Conclusions

High rates of biochar addition to OECD artificial soil and to Kettering loam, a natural soil, induced a subtle level of avoidance behaviour. Effects on survival over a 4-week period were inconsistent, but death and physical damage to some individual earthworms were apparent, and the mechanisms and processes leading to these effects should be investigated further. Earthworm development (mean mass change over time) proved to be a more sensitive measure, revealing negative effects on *L. terrestris* at 10% and 20% (*w*/*w*) wheat biochar applications in OECD soil and at 20% (*w*/*w*) applications of both biochars tested in Kettering loam. The moisture content of *E. fetida* remained remarkably consistent across treatments, indicating that this is not a sensitive measure of effects. The high rates of biochar application resulted in increased water-soluble chloride and phosphate concentrations in simulated soil porewater, both of which could have significant influence on plant growth, microbial activity, invertebrate diversity and nutrient mobility and thus warrant further investigation.

## Electronic supplementary material

ESM 1 (DOCX 853 kb)
