# Supplementary material for: Ecotoxicological assessments of biochar additions to soil employing earthworm species Eisenia fetida and Lumbricus terrestris
Source: Environ Sci Pollut Res Int. 2019 Feb 22;27(27):33410–8. doi: 10.1007/s11356-019-04542-2 (PMC7423795; doi:10.1007/s11356-019-04542-2)
Supplement: Supplementary file 1 — (DOCX 853 kb) [file 11356_2019_4542_MOESM1_ESM.docx]

Supplementary Information - SEM images












Figure S1. Rice Biochar SEM images. Clockwise from top-left: x500, x1000, x2500, x5000 magnification.

V












Figure S2. Wheat Biochar SEM images. Clockwise from top-left: x500, x1000, x2500, x5000 magnification.
